# Oxytocin and testosterone administration amplify viewing preferences for sexual images in male rhesus macaques

**DOI:** 10.1098/rstb.2021.0133

**Published:** 2022-08-29

**Authors:** Yaoguang Jiang, Feng Sheng, Naz Belkaya, Michael L. Platt

**Affiliations:** ^1^ Department of Neuroscience, Perelman School of Medicine, University of Pennsylvania, Philadelphia, PA, 19104, USA; ^2^ Department of Psychology, School of Arts and Sciences, University of Pennsylvania, Philadelphia, PA, 19104, USA; ^3^ Marketing Department, the Wharton School, University of Pennsylvania, Philadelphia, PA, 19104, USA; ^4^ Wharton Neuroscience Initiative, University of Pennsylvania, Philadelphia, PA, 19104, USA; ^5^ School of Management and MOE Frontier Science Center for Brain Science & Brain–Machine Integration, Zhejiang University, Hangzhou, Zhejiang, 310058, People’s Republic of China; ^6^ Champalimaud Center for the Unknown, Lisbon, 1400-038, Portugal

**Keywords:** oxytocin, testosterone, non-human primate, face, sexual skin, sexual attraction

## Abstract

Social stimuli, like faces, and sexual stimuli, like genitalia, spontaneously attract visual attention in both human and non-human primates. Social orienting behaviour is thought to be modulated by neuropeptides as well as sex hormones. Using a free viewing task in which paired images of monkey faces and anogenital regions were presented simultaneously, we found that male rhesus macaques overwhelmingly preferred to view images of anogenital regions over faces. They were more likely to make an initial gaze shift towards, and spent more time viewing, anogenital regions compared with faces, and this preference was accompanied by relatively constricted pupils. On face images, monkeys mostly fixated on the forehead and eyes. These viewing preferences were found for images of both males and females. Both oxytocin (OT), a neuropeptide linked to social bonding and affiliation, and testosterone (TE), a sex hormone implicated in mating and aggression, amplified the pre-existing orienting bias for female genitalia over female faces; neither treatment altered the viewing preference for male anogenital regions over male faces. Testosterone but not OT increased the probability of monkeys making the first gaze shift towards female anogenital rather than face pictures, with the strongest effects on anogenital images of young and unfamiliar females. Finally, both OT and TE promoted viewing of the forehead region of both female and male faces, which display sexual skins, but decreased the relative salience of the eyes of older males. Together, these results invite the hypothesis that both OT and TE regulate reproductive behaviours by acting as a gain control on the visual orienting network to increase attention to mating-relevant signals in the environment.

This article is part of the theme issue ‘Interplays between oxytocin and other neuromodulators in shaping complex social behaviours’.

## 1. Introduction

Facing limitations in environmental and individual resources, organisms must decide how to allocate time and effort to some behaviours at the expense of others [1,2]. Hormones play a well-established role in coordinating trade-offs [3,4] between, for example, mating, pair bonding [5,6], parenting [7,8] and dominance and aggression [9]. In males, neuropeptides such as oxytocin (OT) and arginine vasopressin (AVP), and gonadal androgens such as testosterone (TE), often seem to act in opposition during behavioural regulation [10]. For example, in a wide range of species, including many primates, TE promotes sexual activity and sex-related aggression [9,11] but is downregulated in monogamous as well as paternal males [6,8,12–16]. By contrast, OT facilitates pair-bonding, monogamy and paternal care [6,8,14,16]. Beyond reproduction, TE contributes to aggression, competition and status-seeking behaviours [17–19], whereas OT promotes affiliative behaviours such as trust [20], empathy [21] and mentalizing [22,23].

The behavioural effects of hormones, however, are often context-dependent. For example, while fatherhood is associated with low TE and high OT, sexual activity increases both TE and OT, as does inter-group competition [24–27]. Precisely how different hormones interact with each other to fine-tune behaviour across contexts remains poorly understood [4,10,28], as the neuroendocrinological investigations of neuropeptides and gonadal hormones remain largely parallel efforts and few studies have investigated both sets of compounds in the same behavioural context [28].

Rhesus macaques live in large, hierarchical, mixed-sex groups [1], display complex social behaviours [29,30] and rely heavily on visual displays to communicate [31,32]. In social and mating contexts, both faces and anogenital regions (AGRs, including genitals, anus, upper inner thigh, male scrotum and ischial callosities) of rhesus macaques contain a rich array of information that naturally attracts visual attention [33–35]. Notably, both male and female macaques express red coloration in their faces and rumps [11,13,36,37], and variation in this coloration is salient to members of the opposite sex [38–40]. In females, the coloration of these ‘sexual skins’ intensifies during the mating season due to increased vascular blood flow under the skin surface [37,41], and they tend to be the darkest around ovulation [42,43], thus broadcasting reproductive status to conspecifics. Even though the colour expressed in face and AGR are significantly correlated (for example, in mandrills [44], drills [45] and macaques [46]), it is unclear which area contains more accurate information about the reproductive cycle [42], and which region attracts more attention from conspecifics [39,47]. In male macaques, sexual skins also darken during the mating season [11,13], but it is unclear precisely what these sexual skins signal. In some primates such as mandrills and drills, males with redder faces tend to be higher ranking [45,48,49]. In these species, the male sexual skin is likely to be ‘a badge of status’ signalling the competitive ability of the bearer [11]. In rhesus macaques, however, male sexual skin colour has not been linked definitively to social status [46]. An alternative but not mutually exclusive hypothesis is that male sexual skin is an ‘honest' display of the bearer's TE level and, as high TE is linked to lowered immunocompetence, a male's ability to display costly TE-dependent traits indicates his good health and genetic quality [50].

Beyond coloration, macaque faces also contain other visual cues, such as symmetry, skin quality and secondary sexual features, that convey hormone levels, health, genetic quality and possibly dominance status. All these cues, including coloration, impact potential mate evaluation in humans [51,52] and some are known to shape visual attention in macaque monkeys as well [53–55]. In addition, both faces and AGRs can be used to visually identify a familiar conspecific [1,11]. Thus, faces and AGRs of macaque monkeys contain overlapping yet distinct information, but a direct comparison of visual orienting behaviour when both stimuli are present has never been made, nor has the impact of neuropeptide and steroid hormones on these behaviours ever been examined.

Recent work indicates that socially salient stimuli such as faces and body parts are encoded by neurons in temporal cortex [56–59] and amygdala [60,61]. This information is translated into value signals in the striatum [62], orbitofrontal cortex [35,63] and ventromedial prefrontal cortex [64,65], which then scale the activity of neurons in the visual orienting system to bias attention [66,67]. How this network is ‘tuned' to the behavioural context, such as mate-seeking and intra-sexual competition during the breeding season, or grooming and offspring care outside of the breeding season, remains unknown. One biologically plausible hypothesis is that sex hormones like TE and nonapeptide hormones like OT and AVP differentially upregulate or downregulate specific components of this circuitry to allocate visual attention to the most relevant stimuli, such as the AGR of a potential mate or the face of an offspring, thus facilitating the most adaptive behaviours for the current social context.

Here, we provide an initial, partial test of this hypothesis by directly examining the impact of administering OT and TE on the visual orienting behaviour of male rhesus macaques confronted with images of conspecific faces and AGRs. On each trial, one face and one AGR of the same monkey, drawn from a large image set of familiar and unfamiliar male and female conspecifics, were simultaneously presented to the subject monkey. We hypothesized that: (H1) male monkeys favour AGR images over faces; (H2) male monkeys prefer female images over male images; (H3) OT accentuates male monkeys’ preference for faces whereas TE intensifies their attraction to AGRs; and finally, (H4) male monkeys demonstrate specific selectivity for female AGRs and male faces, respectively, and that OT and TE amplify these biases.

## 2. Results

**Figure 1.**
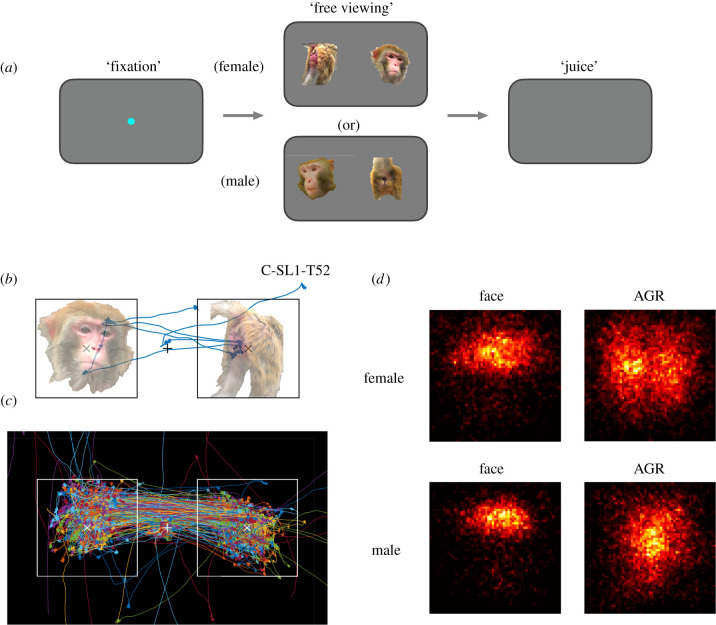
Experimental paradigm and representative gaze patterns. (*a*) The free viewing experiment: at the start of each trial, the monkey fixates on a central fixation spot to initiate the trial. A pair of images, a face and an AGR of the same monkey (male or female), subsequently appear on each side of the screen for 3 s, after which they are replaced by a blank screen and a fixed amount of juice (0.5 ml) is delivered to the subject monkey independent of gaze behaviour during the trial. (*b*) Raw eye movement trace (blue) from one monkey in one example trial depicting fixations (i.e. nodes) and saccades (i.e. traces between nodes). (*c*) Raw eye movement traces from one monkey in one example session (female pictures, 120 trials). Each coloured line represents one trial. Cross: fixation spot; squares: image display windows. (*d*) Population heat maps of all monkeys' gazes in all saline sessions within the female (top row) and male (bottom row) face (left column) and AGR image displays (right column).

First, we examined the spontaneous gaze patterns of male macaques when they were presented with paired images of faces and AGRs of female and male conspecifics (figure 1*a*). We found that male macaques spontaneously oriented gaze towards images of conspecifics (figure 1*b*), and that within a single trial, they shifted gaze back and forth between the two images multiple times (figure 1*c* for an example session, during which the monkey made an average of 11.44 ± 0.17 fixations totalling 2.39 ± 0.02 s within image displays per 3 s trial). Collectively (*n* = 2 male monkeys viewing 360 pairs of female images and 540 pairs of male images), monkeys' gaze tended to focus on the top half of face images but was more evenly distributed across AGRs, and these patterns were consistent for female and male images (figure 1*d*).

**Figure 2.**
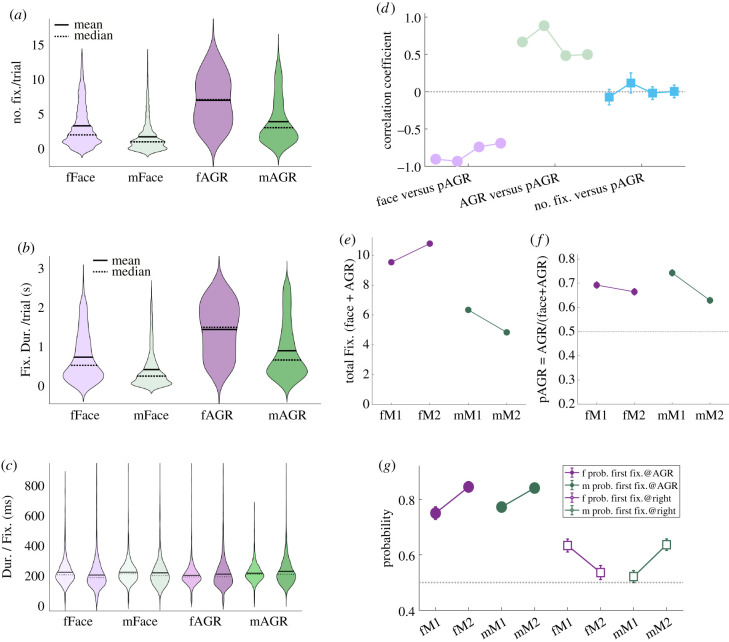
Male monkeys prefer AGRs over faces. (*a*) Male monkeys view AGRs with higher frequency than faces. In addition, male monkeys prefer images of female conspecifics over male conspecifics. *X*-axis, from left to right: female face, male face, female AGR, male AGR. (*b*) Male monkeys view AGRs for longer durations than faces. *X*-axis, from left to right: female face, male face, female AGR, male AGR. (*c*) The distributions of fixation length for 2 subject monkeys (M1 and M2) across four image categories. *X*-axis, from left to right: M1 on female face, M2 on female face, M1 on male face, M2 on male face, M1 on female AGR, M2 on female AGR, M1 on male AGR, M2 on male AGR. (*d*) Proportion of fixations on AGR (pAGR) is negatively correlated with fixations on face (left), positively correlated with fixations on AGR (middle), and not significantly correlated with the total number of fixations (right). Each line has four data points representing in order correlation coefficients for M1 viewing female images, M2 viewing female images, M1 viewing male images and M2 viewing male images. Error bars: estimated coefficient ± 95% confidence interval. (*e*) The total number of fixations is higher for female images. fM1: M1 viewing female images; fM2: M2 viewing female images; mM1: M1 viewing male images; mM2: M2 viewing male images. (*f*) pAGR is constantly above 0.5. fM1: M1 viewing female images; fM2: M2 viewing female images; mM1: M1 viewing male images; mM2: M2 viewing male images. (*g*) The probability of monkeys making the first saccade towards AGR rather than face image is significantly above 0.5, despite small, inconsistent side biases. fM1: M1 viewing female images; fM2: M2 viewing female images; mM1: M1 viewing male images; mM2: M2 viewing male images.

Across all trials, monkeys showed significantly biased orienting towards AGRs over faces (average number of fixations/trial, face = 2.34 ± 0.06, AGR = 5.08 ± 0.09, *p* < 0.0001, Wilcoxon signed-rank) (figure 2*a*). This gaze bias was consistent for both subjects (average number of fixations/trial, M1 face = 2.19 ± 0.09, AGR = 5.43 ± 0.12; M2 face = 2.49 ± 0.09, AGR = 4.73 ± 0.12, *p* < 0.0001, two-way ANOVA). In addition, female images on average attracted more gaze than male images (average number of fixations/trial, female images = 5.08 ± 0.10, male images = 2.79 ± 0.06, *p* < 0.0001, Wilcoxon rank-sum) (figure 2*a*), a trend that was consistent for both subjects (average number of fixations/trial, M1 female images = 4.77 ± 0.14, male images = 3.17 ± 0.10; M2 female images = 5.39 ± 0.14, male images = 2.42 ± 0.08, *p* < 0.0001, two-way ANOVA). Similar biases were observed when we examined overall fixation time instead of number of fixations on each image (overall fixation duration/trial, face = 503.59 ± 13.51 ms, AGR = 1077.90 ± 17.86 ms, female = 1049.8 ± 19.79 ms, male = 618.07 ± 14.26 ms) (figure 2*b*). As is typical for unconstrained viewing, each fixation lasted around 200 ms (mean = 246.65 ms, median = 213.00 ms) (figure 2*c*), and thus number of fixations and total dwell time were highly correlated (*r* = 0.96, *p* < 0.0001). Across subjects, average fixation length did not differ for faces and AGRs, but was significantly longer for male than female images (female images = 225.20 ± 1.19 ms/fixation, male images = 263.18 ± 1.35 ms/fixation, *p* < 0.0001, two-way ANOVA) (figure 2*c*). Henceforth, we will focus analyses on the number of fixations, but will describe changes in total fixation duration and fixation length when relevant.

Because each face image was presented beside an AGR image and *vice versa*, the number of fixations on faces versus AGRs was negatively correlated trial-by-trial (female images: *r* = −0.48, *p* < 0.0001; male images: *r* = −0.17, *p* < 0.0001). We therefore quantified preference for AGR over face images by calculating the proportion of fixations on AGR (pAGR = number of fixations on AGR/(number of fixations on AGR + number of fixations on face)). Naturally, pAGR was negatively correlated with number of fixations on face (female images: *r* = −0.92, *p* < 0.0001; male images: *r* = −0.71, *p* < 0.0001) and positively correlated with number of fixations on AGR (female images: *r* = 0.77, *p* < 0.0001; male images: *r* = 0.51, *p* < 0.0001). Importantly, pAGR was uncorrelated from the total number of fixations made per trial (female images: *r* = −0.00, *p* = 0.950; male images: *r* = 0.03, *p* = 0.344) (figure 2*d*). We thus established two largely independent measures, the total number of fixations (figure 2*e*) and pAGR (figure 2*f*), to quantify male rhesus' gaze attraction to each monkey depicted as well as the relative attractiveness of AGR over face for the same monkey. We also found that the first gaze shift on each trial was biased towards AGRs over faces, regardless of the sex of the monkey depicted (probability of first gaze on AGR, female = 0.78 ± 0.02; male = 0.81 ± 0.01, *p* < 0.0001, *χ*^2^-tests; figure 2*g*).

**Figure 3.**
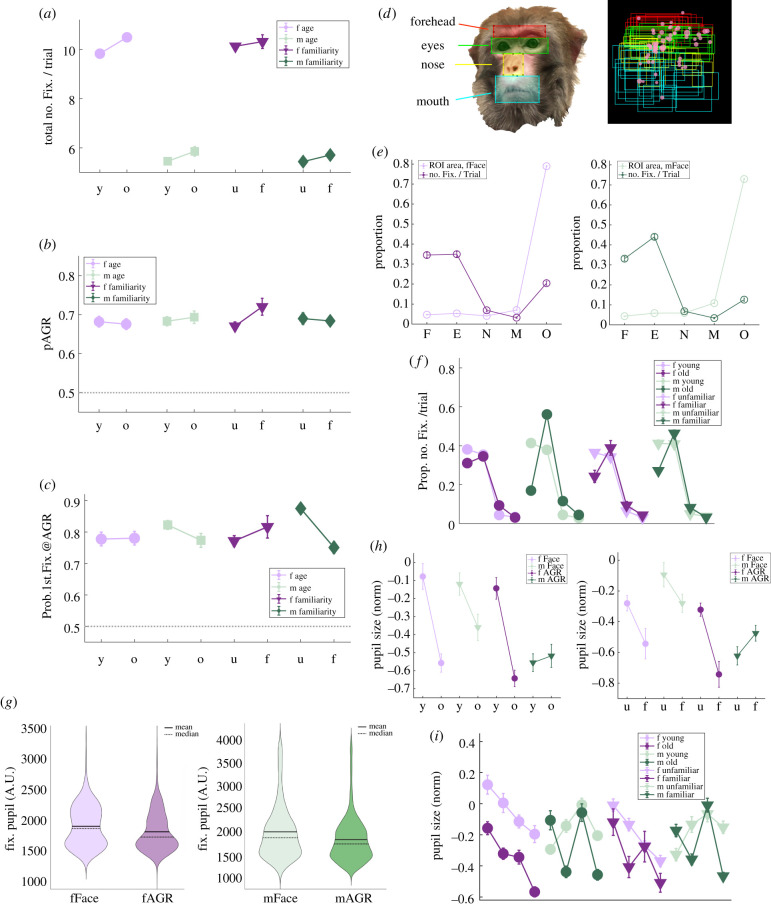
Monkey identity, regions of interest (ROI) and pupil analyses in baseline condition. y, young; o, old; u, unfamiliar; f, familiar. Error bars: mean ± s.e.m. (*a*) Under saline control, male monkeys fixate more on older female and male monkeys. (*b*) This bias towards older conspecifics is reflected only in total number of fixations, not in pAGRs. (*c*) Despite an overall preference for older conspecifics, monkeys' first gaze is more likely to be on AGR rather than face image for young and unfamiliar males. (*d*) Left: an example female face image with 4 ROIs identified. Right: an example set of ROIs for all the face images of the same female monkey, overlaid with one subject monkey's corresponding gaze pattern. Red: forehead; green: eyes; yellow: nose, cyan: mouth. Each pink bubble represents one fixation, with the size of the bubble corresponding to fixation duration. (*e*) Monkeys disproportionally fixate on forehead and eye regions for both female (left) and male (right) faces. F, forehead; E, eyes; N, nose; M, mouth; O, other. (*f*) How monkey identity impacts viewing patterns across different ROIs. Each line has four data points representing in order fixations on forehead, eyes, nose and mouth regions. (*g*) Pupil size during fixation for female (left) and male (right) images. fFace, female faces; fAGR, female AGRs; mFace, male faces; mAGR, male AGRs. (*h*): How monkey identity impacts fixation pupil sizes. (*i*) How monkey identity impacts pupil sizes across different ROIs. Each line has four data points representing in order fixations on forehead, eyes, nose and mouth regions.

Male rhesus monkeys made more fixations on images of older females than on images of younger females (total number of fixations/trial, young female = 9.83 ± 0.17, old female = 10.50 ± 0.15, *p* = 0.001, Wilcoxon rank-sum) and also made slightly more fixations on images of older males than on images of younger males (total number of fixations/trial, young male = 5.46 ± 0.13, old male = 5.86 ± 0.19, *p* = 0.096, Wilcoxon rank-sum). Familiarity of the monkey in the images, by contrast, did not significantly impact total number of fixations (figure 3*a*). Neither age nor familiarity significantly impacted pAGR, although there was a trend towards elevated pAGR for familiar females (pAGR, unfamiliar female = 0.67 ± 0.01, familiar female = 0.72 ± 0.02, *p* = 0.079, Wilcoxon rank-sum) (figure 3*b*). Thus, overall, male macaques were more attentive to the faces and AGRs of older conspecifics compared with younger ones. Although neither age nor familiarity impacted initial gaze shifts on trials with female images, subjects were more likely to visually inspect AGR images first for young and unfamiliar males (probability of first gaze on AGR, young male = 0.82 ± 0.01; old male = 0.77 ± 0.02; unfamiliar male = 0.86 ± 0.02; familiar male = 0.75 ± 0.02, *p* < 0.0001, *χ*^2^-tests) (figure 3*c*).

Macaques are known to orient preferentially to specific features on a face such as the eyes and mouth [31,32,57,58]. We next identified four regions of interest (ROIs) on each face image: forehead, eyes, nose and mouth (figure 3*d*, left for an example ROI map). We found that the male macaques in our study fixated preferentially within these ROIs compared to outside them (figure 3*d*, right for gaze pattern on an example set of face images). Across all trials, for female and male faces alike, monkeys disproportionally fixated on these ROIs, particularly the eyes and forehead (together forehead and eye regions accounted for 69.5 ± 1.2% of fixations on female faces, and 77.2 ± 1.0% of fixations on male faces, despite occupying only 10.0 ± 0.1% and 10.2 ± 0.1% of the total area, respectively) (figure 3*e*). Notably, the forehead regions of young and unfamiliar conspecifics, both male and female, attracted more visual attention than the foreheads of old and familiar ones (percentage of fixations on forehead: young female = 38.1 ± 2.1%, old female = 31.1 ± 1.8%, young male = 41.4 ± 1.5%, old male = 16.9 ± 1.5%, unfamiliar female = 36.5 ± 1.5%, familiar female = 24.2 ± 3.1%, unfamiliar male = 41.2 ± 1.8%, familiar male = 27.2 ± 1.5%, *p* < 0.05, Wilcoxon rank-sums). For eyes, by contrast, those of old and familiar male monkeys were particularly salient (percentage of fixations on eyes: young male = 37.9 ± 1.5%, old male = 56.1 ± 2.1%, *p* < 0.0001; unfamiliar male = 40.9 ± 1.9%, familiar male = 46.3 ± 1.7%, *p* = 0.091, Wilcoxon rank-sum) (figure 3*f*).

We also examined pupil size as an index of attentiveness. Compared with faces, viewing AGR images was associated with smaller pupil sizes (average fixation pupil size, in arbitrary units, female face = 1881.8 ± 11.8; female AGR = 1844.6 ± 11.2; male face = 2133.9 ± 19.2; male AGR = 1974.9 ± 15.7, *p* < 0.0001, one-way ANOVAs) (figure 3*g*), possibly indicating greater covert attention when scanning AGR images. When viewing both female and male faces, as well as female AGRs, pupil sizes were smaller when the images were of old and familiar conspecifics compared to young or unfamiliar conspecifics (average pupil size, *z*-scored against fixation baseline, young female face = −0.08 ± 0.07, old female face = −0.56 ± 0.05; young male face = −0.12 ± 0.06, old male face = −0.36 ± 0.07; young female AGR = −0.14 ± 0.06, old female AGR = −0.64 ± 0.05, *p* < 0.01, Wilcoxon rank-sums; unfamiliar female face = −0.28 ± 0.05, familiar female face = −0.54 ± 0.10; unfamiliar male face = −0.09 ± 0.08, familiar male face = −0.28 ± 0.06; unfamiliar female AGR = −0.32 ± 0.04, familiar female AGR = −0.74 ± 0.08, *p* < 0.05, Wilcoxon rank-sums) (figure 3*h*). For faces specifically, old and familiar female faces generally induced smaller pupil sizes than young and unfamiliar female faces (*p* < 0.0001, Wilcoxon rank-sums) (figure 3*i*), once again suggesting that smaller pupil diameter in this context was indicative of higher attention/vigilance. By contrast, the foreheads of young and unfamiliar males elicited smaller pupils (average pupil size on forehead, *z*-scored against fixation baseline, young male = −0.29 ± 0.03, old male = −0.11 ± 0.06, unfamiliar male = −0.33 ± 0.04, familiar male = −0.17 ± 0.04, *p* < 0.05, Wilcoxon rank-sums), but the eyes of old and familiar males elicited smaller pupils than those of young and unfamiliar counterparts (average pupil size on eyes, *z*-scored against fixation baseline, young male = −0.14 ± 0.06, old male = −0.44 ± 0.07, unfamiliar male = −0.13 ± 0.08, familiar male = −0.36 ± 0.06, *p* < 0.01, Wilcoxon rank-sums) (figure 3*i*).

**Figure 4.**
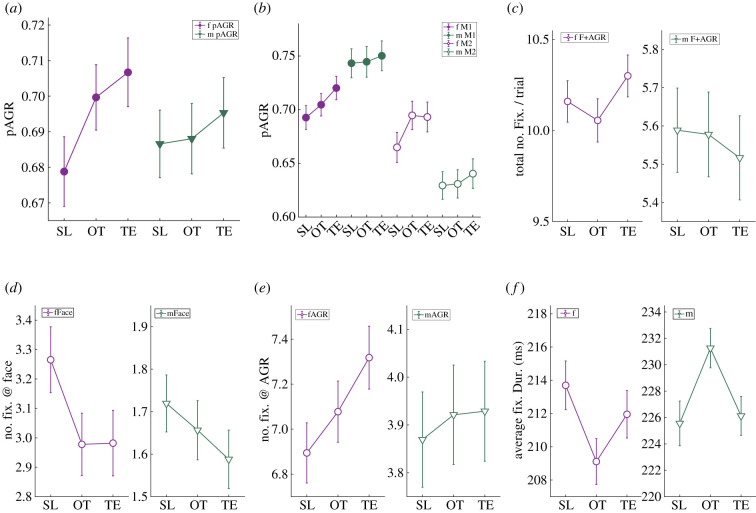
The effects of exogenous OT and TE administration on viewing preferences. Error bars: mean ± s.e.m. (*a*) Compared with SL, OT and TE further enhance male monkeys' bias of favouring AGRs over faces, but only for female pictures. SL, saline. (*b*) This trend for increasing pAGR for female but not male pictures is consistent across monkeys. *X*-axis, from left to right: M1 viewing female images, M1 viewing male images, M2 viewing female images and M2 viewing male images. (*c*) By contrast, neither OT nor TE alters the total number of fixations made towards image displays. (*d*) OT and TE decreased the number of fixations made towards female faces. (*e*) TE but not OT increased the number of fixations made towards female AGRs. (*f*) OT but not TE decreased the fixation length for female pictures and increased fixation length for male images.

We next examined the impact of OT and TE treatment on these patterns. We found that both OT and TE amplified pre-existing preferences for female AGRs over female faces (pAGR, saline (SL) = 0.68 ± 0.01, OT = 0.70 ± 0.01, TE = 0.71 ± 0.01, *p* = 0.098, one-way ANOVA), but did not alter preference for male AGRs relative to male faces (pAGR, SL = 0.69 ± 0.01, OT = 0.69 ± 0.01, TE = 0.70 ± 0.01) (figure 4*a*). These patterns were consistent across both subjects (pAGR, M1 viewing female images, SL = 0.69 ± 0.01, OT = 0.71 ± 0.01, TE = 0.72 ± 0.01; M1 viewing male images, SL = 0.74 ± 0.01, OT = 0.74 ± 0.01, TE = 0.75 ± 0.01; M2 viewing female images, SL = 0.66 ± 0.01, OT = 0.69 ± 0.01, TE = 0.69 ± 0.01; M2 viewing male images, SL = 0.63 ± 0.01, OT = 0.63 ± 0.01, TE = 0.64 ± 0.01) (figure 4*b*). Neither OT nor TE altered the total number of fixations made on images of conspecifics (figure 4*c*), but both decreased the number of fixations made towards female faces at trend level (number of fixations on female face, SL = 3.27 ± 0.11, OT = 2.98 ± 0.11, *p* = 0.063, TE = 2.98 ± 0.11, *p* = 0.096, one-way ANOVA) (figure 4*d*, left). TE but not OT increased the number of fixations made towards female AGRs (number of fixations on female AGR, SL = 6.89 ± 0.13, TE = 7.32 ± 0.14, *p* = 0.029, one-way ANOVA) (figure 4*e,* left). Again, neither OT nor TE altered the number of fixations on male faces or AGRs (figure 4*d*,*e*, right). A three-way ANOVA of image sex (female versus male) by image type (face versus AGR) by drug treatment (SL versus OT versus TE) revealed a significant difference between image sex (female versus male, *p* < 0.0001), image type (face versus AGR, *p* < 0.0001), and a significant interaction between image type and drug treatment (*p* = 0.020), indicating that OT and TE tended to bias visual attention away from faces and towards AGRs. Finally, OT but not TE decreased average fixation length for female pictures and increased fixation duration for male images (average fixation length on female images: SL = 213.70 ± 1.46 ms, OT = 209.11 ± 1.38 ms, *p* = 0.023, on male images: SL = 225.56 ± 1.69 ms, OT = 231.26 ± 1.49 ms, *p* = 0.012, one-way ANOVA) (figure 4*f*).

**Figure 5.**
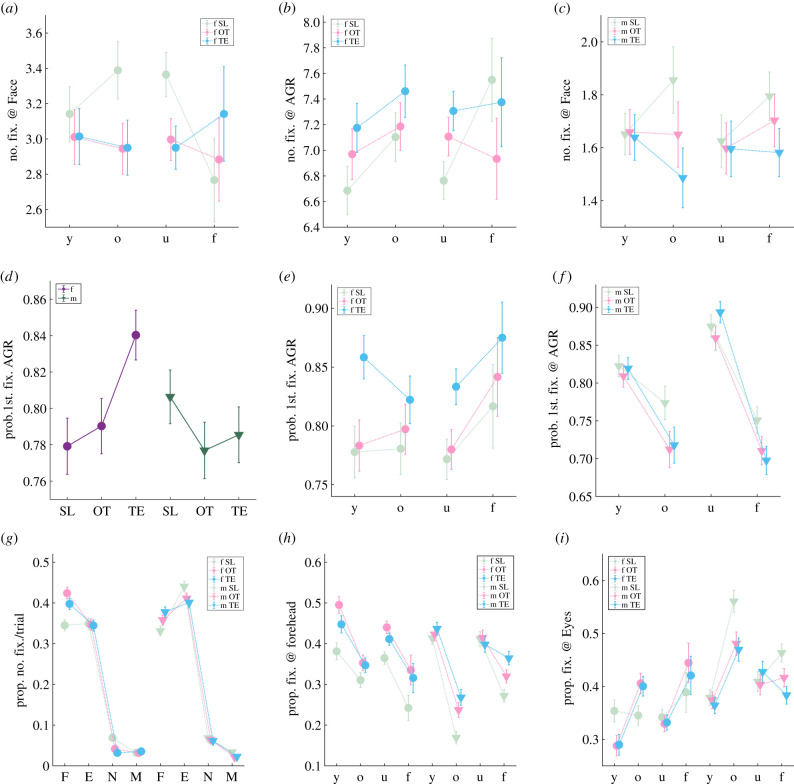
Monkey identity, first gaze and ROI analyses in OT and TE treatment conditions. Error bars: mean ± s.e.m. (*a*) OT and TE decreased the number of fixations made towards old and unfamiliar female faces. (*b*) TE but not OT increased the number of fixations made towards young and unfamiliar female AGRs. (*c*) TE but not OT decreased the number of fixations made towards old and familiar male faces. (*d*) TE but not OT increased the probability of first gaze landing on AGRs for female but not male images. (*e*) TE but not OT specifically increased the probability of first gaze landing on the AGRs of young and unfamiliar females. (*f*) The same effect does not exist when subjects view male pictures. (*g*) Both OT and TE further promoted viewing of forehead regions on female as well as male faces, but they decreased viewing of eye regions for male faces only. (*h*) OT and TE increased forehead viewing for all female faces, but more specifically for old and familiar male faces. (*i*) OT and TE reduced viewing of the eyes, but only for old and familiar male faces.

Upon further examination, we found that both OT and TE decreased the salience of old and unfamiliar female faces (number of fixations on old female faces, SL = 3.39 ± 0.16, OT = 2.94 ± 0.14, *p* = 0.041, TE = 2.95 ± 0.16, *p* = 0.052; on unfamiliar female faces, SL = 3.37 ± 0.13, OT = 3.00 ± 0.12, *p* = 0.033, TE = 2.95 ± 0.12, *p* = 0.018, one-way ANOVAs) (figure 5*a*). Furthermore, TE amplified the salience of young and unfamiliar female AGRs (number of fixations on young female AGRs, SL = 6.69 ± 0.19, TE = 7.18 ± 0.19, *p* = 0.069; on unfamiliar female AGRs, SL = 6.76 ± 0.15, TE = 7.31 ± 0.15, *p* = 0.010, one-way ANOVAs) (figure 5*b*). TE but not OT decreased the number of fixations made on old and familiar male faces (number of fixations on old male faces, SL = 1.86 ± 0.13, TE = 1.49 ± 0.11, *p* = 0.029; on familiar male faces, SL = 1.80 ± 0.09, TE = 1.58 ± 0.09, *p* = 0.098, one-way ANOVAs) (figure 5*c*). TE, but not OT, significantly increased the probability of making the first saccade towards female AGR rather than female faces (probability of first gaze on female AGR, SL = 0.78 ± 0.02, TE = 0.84 ± 0.01, *p* < 0.0001, *χ*^2^-tests) (figure 5*d*), specifically for young and unfamiliar female AGRs (probability of first gaze on young female AGR, SL = 0.78 ± 0.02, TE = 0.86 ± 0.02, *p* < 0.0001; on unfamiliar female AGR, SL = 0.77 ± 0.02, TE = 0.83 ± 0.02, *p* < 0.0001, *χ*^2^-tests) (figure 5*e*). Neither OT nor TE significantly altered the probability of first gaze landing on faces versus AGRs when viewing male images (figure 5*f*).

Within face images, both OT and TE increased attention to the forehead regardless of the sex of the monkey depicted (percentage of fixations, on female forehead: SL = 34.5 ± 1.4%, OT = 42.4 ± 1.4%, *p* = 0.0002, TE = 39.8 ± 1.4%, *p* = 0.013; on male forehead: SL = 33.1 ± 1.2%, OT = 35.8 ± 1.2%, *p* = 0.089, TE = 37.8 ± 1.2%, *p* = 0.031, one-way ANOVAs) (figures 5*g*,*h*). By contrast, both OT and TE decreased attention to the eyes for male face images (figure 5*g*), specifically for old and familiar males (percentage of fixations, on old male eyes: SL = 56.1 ± 2.1%, OT = 48.1 ± 2.2%, *p* = 0.033, TE = 47.0 ± 2.2%, *p* = 0.015; on familiar male eyes: SL = 46.3 ± 2.1%, OT = 41.7 ± 1.8%, *p* = 0.083, TE = 38.4 ± 1.5%, *p* = 0.006, one-way ANOVAs) (figure 5*i*). In summary, both OT and TE increased visual attention to the portion of the face containing sexual skins (i.e. forehead) and decreased attention to the eyes of old and familiar males.

## 3. Discussion

In this study, we used a dual-presentation, free-viewing paradigm to reveal a strong preference for male rhesus macaques to visually inspect the AGRs, rather than faces, of both female and male conspecifics. This was somewhat surprising, as faces are highly salient visual stimuli for rhesus macaques [33,34,68–70] that convey a rich array of information including identity, social status and reproductive state [38,39,47,48]. Monkeys value the opportunity to view conspecific faces so highly that they will forego fluid and food rewards in exchange [33,34]. Similarly, human faces contain a welter of information critical for not only mate evaluation [51,52] but also social interactions in general, such as the potential trustworthiness or competence of another individual [71]. Nevertheless, we found that AGRs are much more potent attractors of attention when presented alongside faces, calling into question the common practice of solely using face images to probe socio-cognitive functions in nonhuman primates, especially in the context of examining the behavioural or neuronal effects of reproductive hormones. Furthermore, in rhesus macaques, faces and AGRs contain redundant information about reproductive state, dominance [46], and, based on our findings, even identity [11]. By selectively attending to faces, monkeys could access not only this information, but also other information solely conveyed by the face, such as symmetry, skin quality and secondary sexual characteristics [53–55]. In this light, we speculate that there are several possible explanations for the pronounced visual bias towards AGR images observerd here: (i) it takes monkeys less time to gather all the information contained within a face image compared with an AGR image, (ii) the natural tendency to visually inspect conspecific faces is attenuated by the need to avoid social conflict, as faces are the source of threat gestures and direct gaze can be considered a sign of aggression in this species [1,30], or (iii) compared with faces, AGR images forecast potential mating opportunities, which are intrinsically rewarding.

Here, we also report that male macaques displayed differential viewing preferences dependent on sex and image type. Overall, monkeys preferably viewed faces as well as AGRs of older monkeys rather than younger ones, regardless of sex. Within faces, however, the forehead regions of young and unfamiliar conspecifics, both male and female, attracted more visual attention than the foreheads of old and familiar ones. For eyes, by contrast, those of old and familiar male monkeys were particularly salient. Together, these results suggest that, when inspecting female images, male rhesus may prioritize the search for reproductive status-related information, which is most prominently displayed on the foreheads of young females and AGRs of old females. When inspecting male images, by contrast, male rhesus may be most concerned with social status and will therefore devote visual attention to old and familiar males, in part because, compared with young adult males (defined as less than 9 years in our sample), older individuals (defined as greater than 8 years in our sample) are more likely to be high-ranking [1,72]. This supposition is supported by the observation that male monkeys were more likely to first check the face of old and familiar males upon image presentation, and that viewing the eyes of old and familiar males was accompanied by more constricted pupils, indicative of vigilance and covert attention [73,74]. Remarkably, our data suggest that male rhesus macaques were able to discriminate male and female, young and old, and familiar and unfamiliar conspecifics solely based on the visual information available not only in face images, but also in AGRs. It is tempting to speculate that such information is broadcast from faces as well as AGRs for adaptive reasons, possibly as a rough map of key social relations in large groups of terrestrial animals moving both towards and away from each other in open environments [11,37].

Finally, we found that both OT, a neuropeptide linked to bonding and affiliation, and TE, a sex hormone implicated in mating and aggression, amplified the pre-existing gaze bias for female AGRs over female faces; neither treatment altered the viewing preference for male images. Because the total number of fixations remained unaltered by hormone treatments, the selectivity of these effects cannot be explained by an overall change in arousal or attentiveness. Instead, OT and TE appear to serve as a gain control for visual processing, a key functional role that both hormones are well suited to play given their broad receptor distributions in the visual orienting network [75–77]. Indeed, a number of studies featuring single-dose OT and TE administration [17,78,79] have demonstrated that the effects of these hormones critically depend on behavioural context and often manifest as amplification of existing biases, as observed here.

It is somewhat surprising that OT and TE impacted visual orienting behaviour in similar ways, especially considering that our original hypothesis was that the neuropeptide OT might bias visual attention towards faces whereas the sex hormone TE could promote viewing of AGRs. There is evidence, however, that OT and TE can act in coordination, especially in reproductive contexts [24,26,80]. For example, both OT and TE are implicated in mate and offspring guarding behaviour [4,13,25–28]. Thus, one possible explanation for our results is that exogenous administration of OT and TE reduce vigilance to potential threats conveyed by conspecific faces [81–85]. The observation that OT and TE both reduced the salience of the eyes of old and familiar male conspecifics supports this hypothesis. It is also possible that both OT and TE shift monkeys into behavioural states that accompany mate evaluation and mate selection [4,28]. This hypothesis is partially supported by the finding that OT and TE both increased gaze towards the foreheads of female and male conspecifics, areas that contain sexual skins. Yet another possibility is that the behavioural effects of administering one hormone may reflect downstream impacts on another hormone. For example, TE has been reported to regulate the expression and binding of neuropeptides, including OT, in brain regions implicated in arousal and visual orienting behaviour (for example, see [86,87]). Finally, OT and TE may also interact with other hormones like AVP [24] to dynamically promote selection of the most appropriate behaviour for the current social and reproductive context.

Our analyses did unveil several significant, albeit somewhat subtle, differences between OT and TE effects, hinting at the possibility that exogenous OT and TE could achieve the same overall behavioural impact via distinctive mechanisms. For example, only TE significantly impacted the number of fixations on female AGRs, and increased the probability of monkeys making an initial gaze shift towards female AGRs rather than faces, mostly for young and unfamiliar females. On the other hand, only OT decreased average fixation duration on female images and increased it for male images. Together these results suggest the possibility that OT biases visual attention away from faces through blunting vigilance to social cues, whereas TE biases gaze towards AGRs by increasing sensitivity to visual cues signalling mating potential, such as sexual skins. Future studies in which OT and TE are delivered in a combinatorial or antagonistic manner can help resolve some of these mechanistic questions. Furthermore, it would be interesting to see if female rhesus macaques naturally display the same visual orienting biases and whether they are similarly impacted by exogenous hormone treatments. Finally, it is also important to evaluate the effects of OT and TE on visual orienting behaviour in a more naturalistic setting, such as monkeys freely viewing videos of conspecifics interacting with each other [35], and compare the results to those obtained in a more controlled experimental paradigm such as ours.

## 4. Methods

### (a) Animals

All procedures reported in this study were approved by the Institutional Animal Care and Use Committee of the University of Pennsylvania, and performed in accordance with their relevant guidelines and regulations. Two male rhesus macaques (M1: L, 15 years old, 11 kg; M2: C, 14 years old, 16 kg) participated in the free viewing experiment and received pharmacological treatments, with 3 days in each treatment condition (SL, OT or TE, see below) for each set of images (9 days in total for female images; 9 days in total for male images).

The face and anogenital region (AGR, or perineum) pictures were taken from 6 female monkeys (B, C, F, HD, HP, SR, 6–23 years old, 7–12 kg) and 9 male monkeys (AM, AR, BR, CN, HK, HL, OK, PN, TM, 6–22 years old, 7–17 kg). For the duration of this experiment, these monkeys lived in two separate colony rooms, with female F and males BR, CN, HL, OK, PN sharing the same room with the subject monkeys. Cages were arranged facing towards the centre of the room, along two walls, permitting all animals to be in continuous visual and auditory contact. This housing arrangement had not been changed for at least a year leading up to the experiment. For the purpose of this experiment, we categorized monkeys living in the same room (and thus having constant visual and auditory contact with each other) as ‘familiar' with each other, and monkeys living in two separate rooms as ‘unfamiliar' with each other. In addition, as skeletal maturity does not occur in macaque monkeys until at least age 8 in.both sexes [88,89], we categorized monkeys younger than 9 years old as ‘young', and those older than 9 years old as ‘old'. The ‘young' age group included all individuals who might still experience developmental changes in the skeletal structure of their faces, whereas the ‘old' group contained only mature individuals who were no longer growing. Finally, for purposes of colony management, most monkeys did not have the opportunity to directly and physically interact with each other, so we did not attempt to infer a linear dominance hierarchy across all animals. As the older monkeys had also lived in the colonies for much longer, however, we inferred that in this group dominance hierarchy roughly correlated with age.

### (b) Pharmacological manipulation

Each free viewing experiment (female or male images) consisted of nine sessions, with each treatment condition (saline/SL, oxytocin/OT or testosterone/TE) repeated three times. The order of treatments was counterbalanced across monkeys (such that in the same week, M1 might receive treatments in the order of OT–TE whereas M2 might receive treatments in the order of TE–OT), as well as within monkeys between weeks (such that M1 might receive treatments in the order of SL–OT in week 1, and OT–SL in week 2), to mitigate any possible order effects. Hormone and saline treatments were delivered on alternating days, with each monkey receiving no more than 2 treatments per week. In each session, approximately 4 h before the free viewing experiment, the subject monkey received either distilled water (2cc, with placebo mixture—see §4d below for detail, on saline control or OT days) or TE (2 mg in 2 cc distilled water) orally (mixed in 15 cc juice) in the home cage. Next, approximately 0.5 h before the behavioural experiment, the subject monkey received either saline (1cc, on saline control or TE days) or OT (25 IU in 1cc saline) via intranasal nebulization in the primate chair.

### (c) Intranasal oxytocin delivery

The procedure for intranasal OT delivery in macaque monkeys has been described in detail previously [68,78,79]. Briefly, monkeys were trained to accept a pediatric nebulizer mask (Pari Labs) over the nose and mouth. Through the nebulizer 1 ml of OT (25 IU ml^−1^ in saline; Agrilabs/Sigma Aldrich) or saline was delivered at a constant rate (0.2 ml min^−1^) over a total of 5 min. Behavioural testing began 30 min after intranasal delivery and continued for 0.5–1 h. The same amount of neuropeptide (25 IU) was delivered to all three monkeys regardless of their weights.

### (d) Oral testosterone delivery

As few studies had administrated exogenous TE in non-human primates, the procedure of oral TE delivery was closely modelled after that in human experiments (for example, see [90–92]). Briefly, for each dose, 2 mg of TE (Sigma Aldrich) was suspended in clear solution with 5 mg of the carrier cyclodextrin, 0.05 ml of 96% ethanol and 2.0 ml of distilled water. The placebo sample was identical to the drug sample only without containing TE. Both TE and placebo were mixed in with juice and delivered through a syringe in the home cage. As prior research has established that the behavioural and physiological effects of TE peak 4 h after oral delivery in humans [90], we administrated behavioural testing within the same time window (3.5–4.5 h post oral delivery).

### (e) Visual stimuli

We photographed male and female macaque monkeys' faces and AGRs in their home cages or primate chairs using the same photography method each time. To compose the picture banks used in this experiment, we took multiple photographs of each individual and selected the most representative 20 face and 20 AGR images for each based on focus, angle, lighting condition, absence of major occlusion etc. Faces with obvious fearful, threatening or appeasing (e.g. lip smacking) expressions were excluded. All the pictures were taken within a two-month period prior to the experiment outside of the theoretical mating season for macaque monkeys. Post-processing of the images was done in Photoshop CC2019 (Adobe Inc.). Irrelevant components such as cage bars, food or toys items inside the cage, head-post implants and primate chair parts were removed. For each monkey, all the face and AGR images were luminance and colour matched using the Photoshop ‘MatchColur’ function, meaning that the average RGB values were consistent within each individual but varied across individuals.

### (f) Experimental set-up

The subject monkey sat in a primate chair (Crist Instruments), with head restrained, in a dark room (luminance approx. 3 cd m^−2^) facing an LCD monitor (BenQ XL2730, 27″, 2560 × 1440, 120 Hz). A computer (Dell Precision Tower 5810, custom built) running Matlab (Mathworks) and Psychtoolbox [93,94] was used to control all aspects of the experiment, including displaying visual stimuli on the monitor, communicating with the eye tracking system (Eyelink, see below), and opening and closing solenoid valves (Christ Instrument) to dispense juice rewards.

During the experiment, the monitor displayed a uniform grey background (luminance approx. 15 cd m^−2^). At the beginning of each trial, a central fixation spot (0.5°, luminance approx. 35 cd m^−2^) came on, and the monkey brought his gaze within the fixation window (3.0° × 3.0°) to initiate image display. Subsequently, a pair of luminance- and colour-balanced images—a face and an AGR of the same monkey—were rendered on each side of the screen for 3 s. Then a blank screen replaced both images, and a fixed amount of juice (0.5 ml) was delivered to the subject monkey. The inter-trial interval was 2–3 s (jittered). The female set consisted of 6 different monkeys, each with 20 pairs of images. The male set consisted of nine different monkeys, each with 20 pairs of images. In each session, one set (female or male) was played in its entirety with each picture displayed once and once only. The order of presentation was randomized across monkey identities as well as images such that in any given session, a face image could be paired with any AGR image of the same monkey and *vice versa*. In addition, which image (face or AGR) was presented on which side (left or right) was also randomized.

After the initial fixation, the subject monkey was free to look anywhere during the 3 s of stimulus presentation as well as the inter-trial interval. Eye movements were recorded with an infrared eye tracking system, Eyelink 1000 Plus (SR Research, primate mount), sampled at 1000 Hz, exported as EDF files, and then pre-processed with a custom Matlab script (Edf2Mat, https://github.com/uzh/edf-converter).

### (g) Data analysis

All data analysis was done in custom Matlab scripts. All statistical tests were two-tailed. For hypothesis testing between two samples, a non-parametric Wilcoxon signed-rank test (for paired samples) or Wilcoxon rank sum test (for un-paired samples) was used. For comparison among more than two samples, an ANOVA was used together with multiple comparisons (Tukey's HSD test) when appropriate. Correlation coefficients were estimated with Pearson's *r*.

## Data Availability

The data are provided in the electronic supplementary material [95].
